# Cerebral Hemodynamic Responses to the Sensory Conflict Between Visual and Rotary Vestibular Stimuli: An Analysis With a Multichannel Near-Infrared Spectroscopy (NIRS) System

**DOI:** 10.3389/fnhum.2020.00125

**Published:** 2020-04-21

**Authors:** Nghia Trong Nguyen, Hiromasa Takakura, Hisao Nishijo, Naoko Ueda, Shinsuke Ito, Michiro Fujisaka, Katsuichi Akaogi, Hideo Shojaku

**Affiliations:** ^1^Department of Otorhinolaryngology, Head and Neck Surgery, Graduate School of Medicine and Pharmaceutical Sciences, University of Toyama, Toyama, Japan; ^2^System Emotional Science Laboratory, Graduate School of Medicine and Pharmaceutical Sciences, University of Toyama, Toyama, Japan; ^3^Department of Otorhinolaryngology, Toyama Red Cross Hospital, Toyama, Japan

**Keywords:** NIRS, sensory conflict, vestibular cortices, temporoparietal junctions, medial superior temporal area, intraparietal sulcus

## Abstract

Sensory conflict among visual, vestibular, and somatosensory information induces vertiginous sensation and postural instability. To elucidate the cognitive mechanisms of the integration between the visual and vestibular cues in humans, we analyzed the cortical hemodynamic responses during sensory conflict between visual and horizontal rotatory vestibular stimulation using a multichannel near-infrared spectroscopy (NIRS) system. The subjects sat on a rotatory chair that was accelerated at 3°/s^2^ for 20 s to the right or left, kept rotating at 60°/s for 80 s, and then decelerated at 3°/s^2^ for 20 s. The subjects were instructed to watch white stripes projected on a screen surrounding the chair during the acceleration and deceleration periods. The white stripes moved in two ways; in the “congruent” condition, the stripes moved in the opposite direction of chair rotation at 3°/s^2^ (i.e., natural visual stimulation), whereas in the “incongruent” condition, the stripes moved in the same direction of chair rotation at 3°/s^2^ (i.e., conflicted visual stimulation). The cortical hemodynamic activity was recorded from the bilateral temporoparietal regions. Statistical analyses using NIRS-SPM software indicated that hemodynamic activity increased in the bilateral temporoparietal junctions (TPJs) and human MT+ complex, including the medial temporal (MT) area and medial superior temporal (MST) area in the incongruent condition. Furthermore, the subjective strength of the vertiginous sensation was negatively correlated with hemodynamic activity in the dorsal part of the supramarginal gyrus (SMG) in and around the intraparietal sulcus (IPS). These results suggest that sensory conflict between the visual and vestibular stimuli promotes cortical cognitive processes in the cortical network consisting of the TPJ, the medial temporal gyrus (MTG), and IPS, which might contribute to self-motion perception to maintain a sense of balance or equilibrioception during sensory conflict.

## Introduction

Stability of self-motion perception is obtained through a composite of multimodal sensory inputs such as visual and nonvisual (e.g., vestibular and proprioceptive) information (Butler et al., 2010; Fetsch et al., 2010). Sensory mismatch (sensory conflict) among different sensory information during body motion induces vertigo and instability of posture (Brandt, 1999) as well as motion sickness and an undesirable illusion of body movements (Brandt, 1999; Keshavarz et al., 2015).

In mammalian species, the vestibular system in the inner ear has two sets of receptors, the semicircular canals and the otoliths (the utricle and saccule), which together sense angular and linear acceleration of the head, respectively, in three dimensions (Smith, 2017). This sensory system is critical to orientation and locomotion: the vestibular system is essential to maintain stable vision during unintentional head movements by generating rapid compensatory eye movements [i.e., the vestibulo-ocular reflexes (VORs)] that maintain stable visual images of the world in the retina (Smith, 2017). Acute elimination of unilateral vestibular inputs due to vestibular neuritis induces sudden ataxia and disturbance of postural stability (Horak, 2009; Peterka et al., 2011). It is suggested that sensory conflict is induced by differences between visual and vestibular information, where each information signal represents different spatial representations of the body and head based on stored experiences (Reason, 1978; Oman, 1982). Thus, coherent integration of multisensory inputs, especially visual and vestibular cues, is essential for appropriate self-motion perception.

Monkey neurophysiological studies reported visual and vestibular integration in several cortical regions including: (1) the dorsal medial superior temporal (MST) area that processes optic flow to induce motion and self-motion perception (Duffy, 1998; Gu et al., 2008); (2) the ventral intraparietal (VIP) area, in which neurons respond to visual and vestibular inputs and are sensitive to visual heading (Chen et al., 2011); and (3) the visual posterior Sylvian (VPS) area located at the posterior edge of the Sylvian fissure, in which neurons responded dominantly to vestibular inputs (Chen et al., 2011). Multisensory information including visual, vestibular, and proprioceptive signals also converges on the parieto-insular vestibular cortex (PIVC), which is essential for vestibular information processing (Guldin et al., 1992; Lewis and Van Essen, 2000).

Human functional magnetic resonance imaging (fMRI) studies using galvanic or caloric stimulation (Bucher et al., 1998; Fasold et al., 2002) reported that vestibular stimuli activated the regions involved in information processing of optic flow including the MST and VIP, suggesting that visual and vestibular information are converged and integrated in these cortical areas. Furthermore, various vestibular stimulations (e.g., caloric and galvanic stimulations as well as those to elicit vestibular evoked myogenic potentials) activated the posterior and anterior insula, temporoparietal junction (TPJ), posterior parietal cortex (PPC), somatosensory cortex, and other brain regions (Lopez and Blanke, 2011). The TPJ, which is a wide cortical region including the posterior superior temporal gyrus (pSTG), angular gyrus (AG), supramarginal gyrus (SMG), and the parietal operculum, receives multimodal information including vestibular as well as somatosensory and visual inputs (zu Eulenburg et al., 2012; Bzdok et al., 2013). The TPJ has been implicated in multimodal integration such as visual–vestibular interactions (Pfeiffer et al., 2014). Thus, the MST, the TPJ, and the PPC including the VIP might be crucial for the integration of visual and vestibular information in humans. However, it is unclear how these regions work during sensory conflict between visual and vestibular inputs in humans.

fMRI, positron emission tomography (PET), and magnetoencephalography (MEG) have often been used to investigate various human cognitive brain functions. However, these imaging techniques have a fundamental problem when applied to research on visual and vestibular integration: natural vestibular stimulation is usually induced by rotatory or linear acceleration movements of the subject’s head, whereas fMRI, PET, and MEG require movements of the subject’s head to be restricted (see below).

Recent studies using fMRI explored human brain activations when visual and vestibular cues were either complementary or in conflict (Roberts et al., 2017; Schindler and Bartels, 2018). Roberts et al. (2017) used horizontal optokinetic (visual) stimulation of black and white stripes and caloric (vestibular) stimulation; however, vestibular stimulation was artificial and without head movements because of the high restrictiveness of fMRI for head rotation. Schindler and Bartels (2018) used a special aircushion placed inside a head coil for fMRI, and subjects voluntarily rotated their heads from center to either approximately +30° or approximately −30° as vestibular stimulation. In this case, the vestibular stimulation was not artificial; however, the speed and angle of the rotation were not constant and were uncontrolled. Thus, fMRI is not suitable for experiments on vestibular stimulation because of the restricted movement of the head required during examination.

To circumvent these limitations, functional near-infrared spectroscopy (fNIRS) was used to investigate cortical hemodynamic responses during sensory conflict between visual and horizontal rotatory (vestibular) stimulations. fNIRS is a functional neuroimaging technique to detect task-related cortical activation by measuring oxygenated hemoglobin (Oxy-Hb) and deoxygenated hemoglobin (Deoxy-Hb) in the brain (Jöbsis, 1977; Colacino et al., 1981). The fNIRS system is more compact and robust against a subject’s motion compared with fMRI, PET, and MEG and consequently is more suitable for analysis of task-related cortical activity during motion (Mihara et al., 2008; Takakura et al., 2015). fNIRS has been used to study visual and vestibular integration in previous studies. Some researchers investigated hemodynamic activity during the control of postural balance in computed dynamic posturography (CDP), which can create a sensory conflict situation among the visual, vestibular, and somatosensory inputs artificially, using a multichannel Near-Infrared Spectroscopy (NIRS) system (Karim et al., 2013; Takakura et al., 2015; Lin et al., 2017). These studies suggested that the TPJ including the SMG and superior temporal gyrus (STG), premotor cortex, and supplementary motor area might be involved in visual and vestibular integration and postural control during CDP. However, these studies mainly applied linear acceleration (i.e., otolith stimulation) as the vestibular stimulation, whereas natural rotatory acceleration (i.e., semicircular canal stimulation) has not been used to investigate visual vestibular integration in previous human studies. We herein investigated cortical hemodynamic activities during sensory (vestibular and visual) integration in congruent and incongruent spinning paradigms using a rotatory chair and portable NIRS systems.

We hypothesized that vestibular and visual stimuli with sensory conflict would activate the cortical regions in and around the TPJ including the bilateral upper parts of the temporal lobe, the parietal lobe, and posterior parts of the frontal lobe. In the present study, to investigate cortical activity elicited by sensory conflict between rotatory vestibular stimuli (rotation of the body) and rotatory visual stimuli (moving white stripes on a screen surrounding a subject), we analyzed hemodynamic activity in and around the TPJ in the congruent condition without sensory conflict, in which visual stripes moved opposite the rotatory direction of the body, and the incongruent condition with sensory conflict, in which visual stripes moved in the same rotatory direction of the body.

## Materials and Methods

### Subjects

Fourteen healthy men [aged 25.8 ± 8.2 (mean ± SD) years, all right-handed] participated in this study. None of the subjects had a medical history of ear diseases, vertigo, or head injury. They were treated in accordance with the Declaration of Helsinki and the U.S. Code of Federal Regulations for the protection of human subjects. Written consent was obtained from each subject, and the experiments were conducted according to a protocol approved by the ethical committee for human experiments of the University of Toyama.

In the present study, subjects sat on a chair that rotated to the left or right, and moving white stripes were projected on a screen in front of them. Portable NIRS systems were set on the backrest of the chair to record cortical hemodynamics. Head angular velocity and its acceleration/deceleration as vestibular stimulation were controlled by rotating the chair, whereas the stripes moved in two different conditions. In the congruent condition, the stripes moved in the opposite direction of chair rotation (natural visual stimulation), whereas in the incongruent condition, the stripes moved in the same direction of chair rotation (conflicted visual stimulation).

### Setup and Tasks

**Figure 1:**
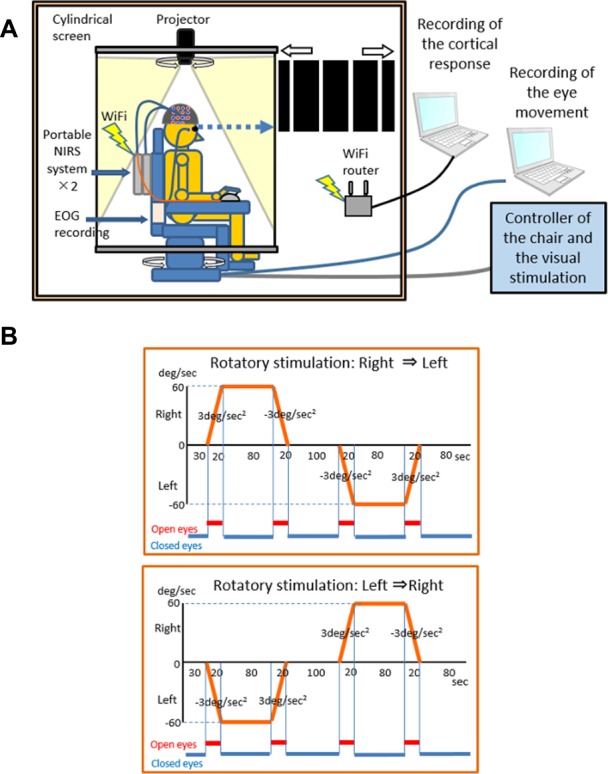
An illustration of the laboratory instruments used in the experiment. The experimental system consists of two portable NIRS systems, an EOG recording unit, a rotary chair for rotary stimulation, a cylindrical screen, and a projector for OKN stimulation **(A)** and the rotatory stimulation protocol **(B)**.

In the present experiment, a visual–vestibular stimulator (OKN/VOR stimulator^®^; First Medicals Co. Ltd., Tokyo, Japan) consisting of a rotatory chair, cylindrical screen (diameter: 150 cm), and projector was used (Figure 1A). The axis of rotation of the rotatory chair was matched to the center of the cylindrical screen. Black (visual angle: 27.5°) and white (visual angle: 2.5°) stripes were projected on the screen. The stimulator could set and control the angular velocity, acceleration, and deceleration of the rotatory chair and specify the direction of horizontal movements of the stripes, which was identical or opposite that of the rotatory chair, at the same speed. Each subject sat on the rotatory chair and kept his eyes closed except when he was requested to open his eyes. The timing of opening and closing of eyes was instructed by sounds, and thus, we could not use noise-cancelling earphones or earplugs. Thirty seconds after the onset of the task, the rotatory chair was accelerated to rotate to the left or right side at 3°/s^2^ for 20 s and then rotated at a constant angular velocity (60°/s) for 80 s. After the constant rotation, the rotatory chair decelerated at 3°/s for 20 s and stopped for 100 s. Then, the rotatory chair was accelerated to the opposite side at 3°/s for 20 s, rotated in a constant angular velocity (60°/s) for 80 s, decelerated at 3°/s^2^ for 20 s, and stopped for 80 s. Each subject was requested to open his eyes during acceleration or deceleration periods for 20 s to watch the movement of the strips (i.e., visual stimulations) projected on the cylindered screen. There were two kinds of the rotatory stimulations [a “right to-left” task (right rotation followed by left rotation) and a “left-to-right” task (left rotation followed by right rotation; Figure 1B)]. Each task included four acceleration/deceleration phases consisting of one acceleration phase to the right, one deceleration phase to the right, one acceleration phase to the left, and one deceleration phase to the left.

Two kinds of visual stimulation were applied. In the “congruent” visual stimulation, the stripes were accelerated or decelerated in the opposite rotatory direction of the rotatory chair at 3°/s^2^ relative to the subject’s head. In the “incongruent” visual stimulation, the stripes were accelerated or decelerated in the same rotatory direction of the rotatory chair at 3°/s^2^ relative to the subject’s head. We analyzed hemodynamic responses in the acceleration phases. Thus, there were four experimental conditions based on a combination of acceleration direction of the rotatory chair and visual stimulation: condition 1 (acceleration to the right and congruent visual stimulation), Condition 2 (acceleration to the left and congruent visual stimulation), Condition 3 (acceleration to the right and incongruent visual stimulation), and Condition 4 (acceleration to the left and incongruent visual stimulation). Each rotation task (i.e., “right-to-left” or “left-to-right” task in Figure 1B) was pseudo-randomly repeated four times in the congruent and incongruent visual stimulations, resulting in a total of four trials for each condition.

Self-assessment of the strength of an uncomfortable vertiginous sensation during the acceleration phase was performed after each rotation task using a visual analog scale (VAS), and mean VAS scores in the congruent and incongruent visual stimulations were calculated for each subject.

### fNIRS Recording

Two portable continuous-wave (CW) fNIRS imaging systems (LIGHTNIRS^®^; Shimadzu Co., Ltd., Kyoto, Japan) were attached firmly on the backside of the backrest of the rotatory chair and used to measure cerebral hemodynamics (Figure 1A). LIGHTNIRS^®^ has eight light sources and eight light detectors in one system. This commercial portable fNIRS system allows the use of two LIGHTNIRS^®^ systems as one synchronized CW fNIRS system with 16 light sources and 16 light detectors by connecting the two systems with a SYNC cable. The lights at three different wavelengths (780, 805, and 830 nm) with a pulse width of 5 ms were emitted from the light-source optodes, and the lights were detected by the light-detector optodes. Signals from the light-detector optodes were processed based on a modified Lambert–Beer law to measure changes in Hb concentration [Oxy-Hb, Deoxy-Hb, and Total-Hb (Oxy Hb + Deoxy Hb)] (Seiyama et al., 1988; Wray et al., 1988).

**Figure 2:**
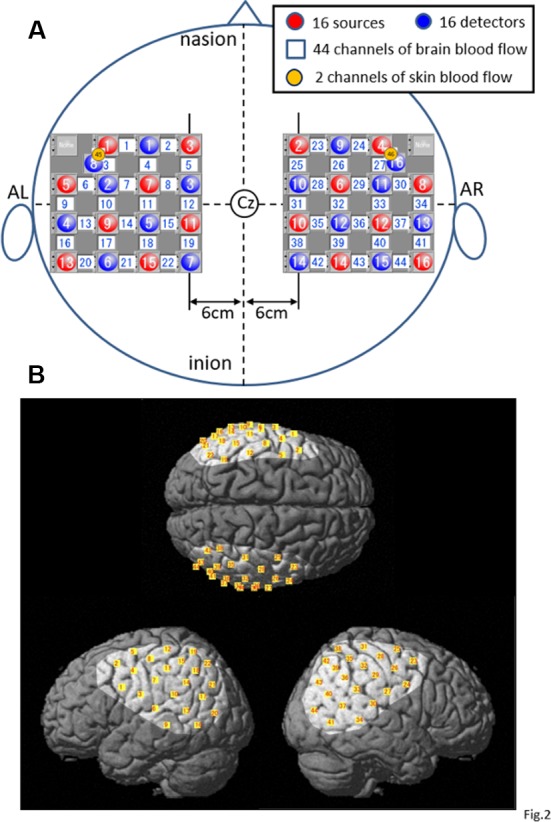
Location of the NIRS optodes. **(A)** The arrangement of the optodes (sources and detectors) and recording channels. AL, left preauricular position; AR, right preauricular position; Cz, vertex in 10–20 EEG recording methods. **(B)** Highlighted areas indicate the recorded cortical regions covered with the optodes in the present study. Yellow small squares indicate the averaged coordinates of the NIRS channels among all subjects.

After the subject sat on the chair, his body was tightly fixed in the chair to prevent falling during rotation of the chair, and he was fitted with a head cap for NIRS recording (FLASH-PLUS; Shimadzu Company Limited). The vertex position of the head cap was positioned at the vertex (Cz) in the 10–20 EEG system, and the 16 light-source optodes and 16 light-detector optodes were placed on the head cap, which has NIRS probe holders (Figure 2A), and the optodes were positioned crosswise from each other.

A 4 × 4 square arrangement of probe holders was used and set on the bilateral temporoparietal areas of the head as follows: a horizontal line connecting the probes and channels in the most upper part of the square holder was set 6 cm lateral from the sagittal midline of the head connecting the nasion, Cz, and inion, whereas the line running vertically through the center of the square holder was set to align with the coronal midline of the head connecting the right and left preauricular positions (AR and AL, respectively) and Cz in each hemisphere (Figure 2A).

In the present study, the 15 detector optodes were placed 3 cm away from the 15 source optodes. The midpoints between the source and detector optodes were called “NIRS channels,” which resulted in 44 channels in total. NIRS signals from the light-detector optodes are supposed to reflect hemodynamic activity under these channels. However, these NIRS signals (whole signals) include not only intracerebral (cerebral cortex) but also extracerebral (scalp, skull, and cerebrospinal fluid) components of hemodynamic activity (Niederer et al., 2008; Ishikuro et al., 2014). Therefore, another two detector optodes were placed 1.5 cm away from the source optodes to record the extracerebral signals (Niederer et al., 2008; Ishikuro et al., 2014), resulting in two channels and corresponding signals (Figure 2A). The probe holes at the anterior–inferior corner of the bilateral 4 × 4 probe holders were not used in both hemispheres. Three-dimensional coordinates of the optodes were measured by a 3-D digitizer (Nirtrack, Shimadzu Co., Limited; Takakura et al., 2011; Ishikuro et al., 2014; Nakamichi et al., 2018).

To determine the anatomical locations of the NIRS channels, we used the “spatial registration of NIRS channel locations” function of the NIRS-SPM (statistical parametric mapping) Version 4_r1 software, which is an SPM5- or SPM8- and MATLAB-based software package for the statistical analysis of NIRS signals (Ye et al., 2009; downloadable from https://bispl.weebly.com/nirs-spm.html). Using the “stand-alone” option (without using MRI images), we estimated the locations of the NIRS channels on the normalized brain surface (Friston et al., 1995; Ashburner et al., 1997; Ashburner and Friston, 1999) using a Montreal Neurological Institute (MNI) brain template, which corresponds to the space identified by Talairach and Tournoux (1988). In each subject, the estimated locations of the NIRS channels were labeled using the 3-D digital brain atlas (Talairach daemon; Lancaster et al., 2000), which is incorporated into the NIRS-SPM. The averaged locations of the NIRS channels (yellow small squares) and covered cortical areas (highlighted on the standard brain) across all subjects are indicated on the standard brain in Figure 2B. The recording cortical areas included the bilateral ventral part of the supraparietal lobule (vSPL), infraparietal sulcus (IPS), SMG, AG, pSTG, parietal operculum (p-OP), frontal operculum (f-Op), ventral part of the precentral and postcentral gyrus (PrG and PoG), posterior middle temporal gyrus (pMTG), and ventral third visual association area (V3; Figure 2B).

### Data Analysis

#### Analysis of Subjective Vertiginous Sensation

Shapiro–Wilk tests, initially performed to check normality of the distribution of the VAS scores in the congruent and incongruent visual stimulations, indicated normal distribution in the congruent visual stimulation (*p* = 0.058) and non-normal distribution in the incongruent visual stimulation (*p* = 0.023). Therefore, we applied nonparametric statistical analyses to the mean VAS scores: the Wilcoxon sign rank test was performed to compare the mean VAS scores between the congruent and incongruent visual stimulations. We also analyzed the correlation between the VAS scores and hemodynamic activity in the congruent and incongruent visual stimulations using Spearman’s rank coefficient test.

#### Analysis of Hemodynamic Responses

The NIRS data consisted of four trials for each condition because each rotation task (i.e., “right-to-left” or “left-to-right” task in Figure 1B) was pseudo-randomly repeated four times in the congruent and incongruent visual stimulations. We analyzed increases in Oxy-Hb concentration and decreases in Deoxy-Hb concentrations as increases in neural activity because typical hemodynamic responses to neural activation consist of an increase in Oxy-Hb (Hoshi et al., 2001; Strangman et al., 2002; Yamamoto and Kato, 2002) and a decrease in Deoxy-Hb (Zhang et al., 2016, 2017) and amplitudes of Oxy-Hb signals are larger than those of Deoxy-Hb signals (Sato et al., 2016).

We analyzed only the data obtained during the acceleration periods (i.e., Conditions 1–4) and not that obtained during the deceleration periods, because our preliminary study indicated no significant hemodynamic responses during the deceleration periods. First, we computed the cerebral component of the NIRS signals by a simple-subtraction method (Schytz et al., 2009; Nakamichi et al., 2018), where the cerebral hemodynamic activity = (whole signals) minus (the extracerebral signals) located nearest to given whole signals. A band-pass filter (0.01–0.1 Hz) was used to eliminate long-term baseline drift and higher-frequency cardiac or respiratory activity from the cerebral component of the NIRS signals (Cordes et al., 2001; Lu et al., 2010). Second, to analyze the temporal changes of hemodynamics, the NIRS data for Oxy-Hb and Deoxy-Hb concentrations were summed and averaged for the onset of 20 s of acceleration in all conditions. The averaged responses were corrected for baseline activity from −10 to 0 s.

We also performed group analyses of the NIRS data based on the general linear model (GLM) using NIRS-SPM software^1^ (Ye et al., 2009). After the subtraction and filtering (see above), we initially extracted two long data measurements, one each during the congruent and incongruent visual simulations, in each subject. The long data measurement in the congruent stimulation included the data in Condition 1 (acceleration to the right and congruent visual stimulation), Condition 2 (acceleration to the left and congruent visual stimulation), Deceleration Condition 1 (deceleration to the right and congruent visual stimulation), and Deceleration Condition 2 (deceleration to the left and congruent visual stimulation), whereas that in the incongruent stimulation included the data in Condition 3 (acceleration to the right and incongruent visual stimulation), Condition 4 (acceleration to the left and incongruent visual stimulation), Deceleration Condition 3 (deceleration to the right and incongruent visual stimulation), and Deceleration Condition 4 (deceleration to the left and incongruent visual stimulation). NIRS data in the congruent and incongruent stimulations were separately analyzed in each subject using GLM NIRS-SPM software for each acceleration condition (i.e., Conditions 1 and 2 in congruent visual stimulation and Conditions 3 and 4 in incongruent visual stimulation). In the GLM analyses, the rotatory acceleration periods with optokinetic visual stimulation were defined as the task periods, whereas the periods with no vestibular and no visual stimulation (periods with rotation at constant angular velocity with eyes closed and periods with no rotation and eyes closed) were defined as the baseline periods. Then, group statistical analyses were performed in each condition using the NIRS-SPM. The resultant *T*-statistic maps were superimposed on the standardized MNI brain in each condition. The statistical significance level was set at *p* < 0.05 as corrected by the false discovery rate (FDR; Benjamini and Hochberg, 1995).

### Correlation Analysis Between Hemodynamic Cortical Activity and Subjective Vertiginous Sensation

We analyzed the correlation between hemodynamic activity and the strength of subjective vertiginous sensation (VAS scores) in each condition. Mean VAS scores in the congruent visual condition were used for the correlation analyses in Conditions 1 and 2, and those in the incongruent visual condition were used in the analyses in Conditions 3 and 4. Spearman’s rank coefficient test between *T*-values and mean VAS scores was performed for all MNI coordinates in each condition, and then *p*-value maps were superimposed on the standardized brain (MNI coordinate system). The statistical significance level was set at *p* < 0.05.

## Results

### Statistical Analysis of Subjective Vertiginous Sensation

**Figure 3:**
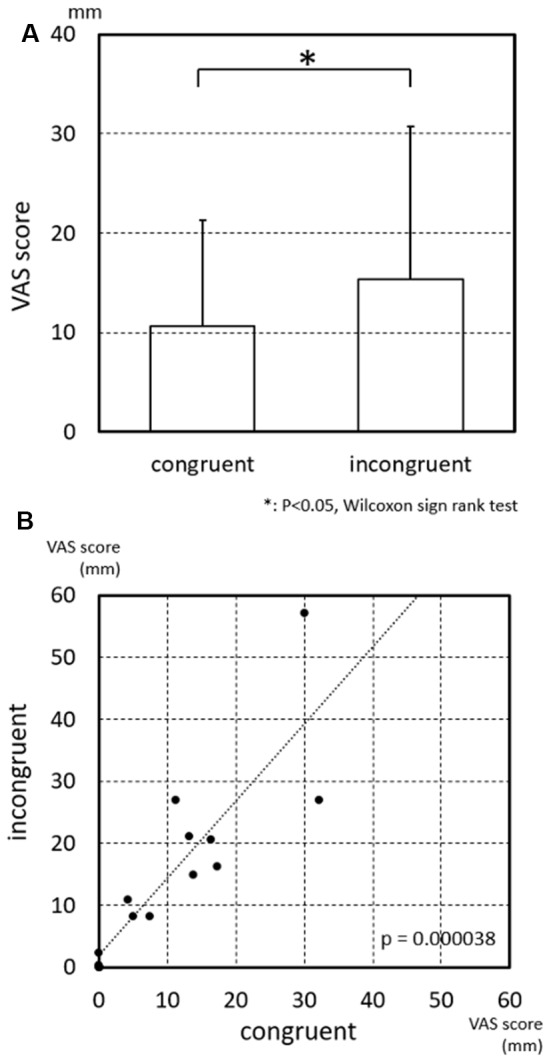
Visual analog scale (VAS) scores of subjective vertiginous sensation in the congruent and incongruent conditions. **(A)** Comparison of VAS scores of subjective vertiginous sensation between the congruent and incongruent conditions. Subjective vertiginous sensation in the incongruent condition is significantly larger than that in the congruent condition by Wilcoxon sign rank test. Error bars indicate the standard deviation. *p < 0.05. **(B)** Relationships of VAS scores of subjective vertiginous sensation between the congruent and incongruent conditions. A positive significant correlation of the strength of subjective vertiginous sensation is found between congruent and incongruent conditions with Spearman’s rank coefficient test, with *R* = 0.877353 and *p* = 0.000038.

The results of the statistical analysis indicated that the strength of subjective vertiginous sensation was significantly larger in the incongruent than congruent conditions (Figure 3A; *p* = 0.030, Wilcoxon sign rank test). Furthermore, the VAS scores in the incongruent condition were significantly and positively correlated with those in the congruent condition (Figure 3B; *p* = 0.000038, Spearman’s rank coefficient test), suggesting that the sensitivity of the subjects to the visual and vestibular stimulations to evoke vertigo was heterogeneous.

### Hemodynamic Activity in Each Condition

**Figure 4:**
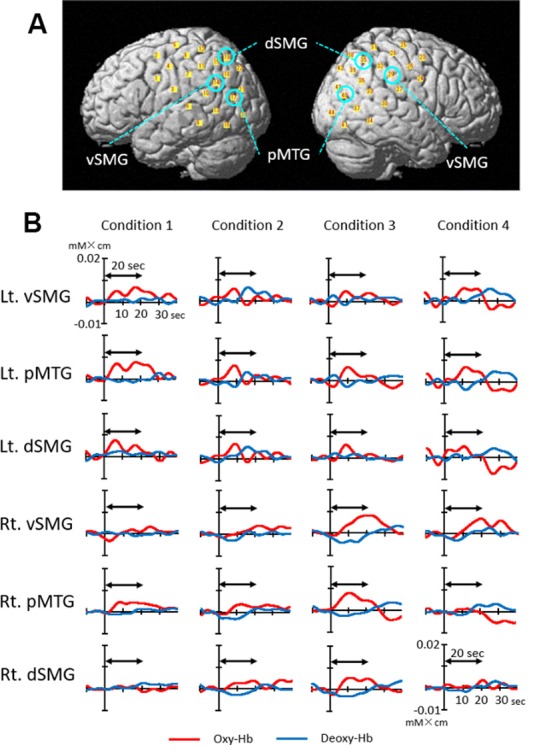
Examples of cerebral hemodynamic activity in the bilateral ventral part of the supramarginal gyrus (vSMG), posterior part of the middle temporal gyrus (pMTG), and dorsal part of the SMG (dSMG). **(A)** Three-dimensional locations of six channels presented in B are indicated. **(B)** Cerebral hemodynamic activity during Condition 1 (congruent visual and right rotatory stimulation), Condition 2 (congruent visual and left rotatory stimulation), Condition 3 (incongruent visual and right rotatory stimulation), and Condition 4 (incongruent visual and left rotatory stimulation) is shown. Red and blue lines indicate Oxy-Hb and Deoxy-Hb, respectively. The data are derived from the same subject. The two-way arrow indicates the rotation period for 20 s. Rt., right; Lt., left.

Figure 4A depicts the 44 channel positions set on the bilateral temporoparietal cortical areas in a representative subject. Figure 4B shows the event-triggered average waveforms of Oxy-Hb and Deoxy-Hb of each channel in the bilateral ventral part of the SMG (vSMG), pMTG, and dorsal part of the SMG (dSMG) in each condition in a representative subject. Both increases in Oxy-Hb and decreases in Deoxy-Hb during the task period were observed in the left vSMG and bilateral pMTG in Condition 1; bilateral pMTG and right dSMG in Condition 2; bilateral pMTG, right vSMG, and dSMG in Condition 3; and bilateral vSMG and left pMTG in Condition 4. These results indicated that the activated cortical areas were different depending on the conditions.

**Figure 5:**
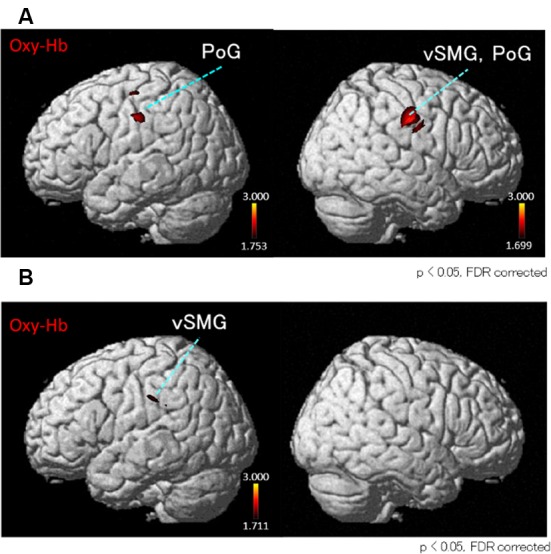
*t*-Statistical maps of the group statistical analyses in Oxy-Hb data using NIRS-SPM in conditions with “congruent” visual stimulation. **(A)** In Condition 1 (acceleration to the right), small cortical regions in the bilateral ventral part of the postcentral gyrus (PoG) and the right ventral part of supramarginal gyrus (vSMG) are activated. **(B)** In Condition 2 (acceleration to the left), small cortical regions in the left vSMG are activated.

**Figure 6:**
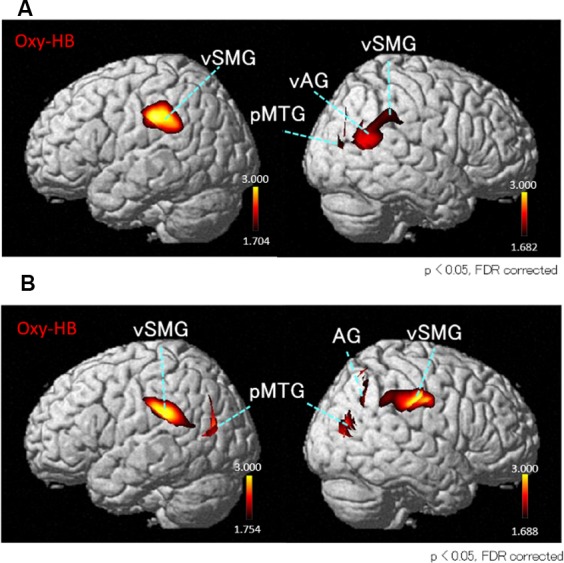
*T*-statistical maps of the group statistical analyses in Oxy-Hb data using NIRS-SPM in conditions with “incongruent” visual stimulation. **(A)** In Condition 3 (acceleration to the right), cortical regions in the bilateral ventral parts of the vSMG, right ventral part of the angular gyrus (vAG), and right posterior part of the middle temporal gyrus (pMTG) are activated. **(B)** In Condition 4 (acceleration to the left), cortical regions in the bilateral vSMG, right AG, and bilateral pMTG are activated.

Next, we performed the group analyses of the Oxy-Hb and Deoxy-Hb data using NIRS-SPM in each condition. However, the group statistical analyses of NIRS Deoxy-Hb data did not indicate significant changes (data not shown). The results of the group statistical analyses of NIRS Oxy-Hb data are shown in Figures 5, 6 (side view). The statistical results are also listed in Table 1. The topographical maps indicated significant activation in the left ventral primary somatosensory area (S1) and the right vSMG under Condition 1 (Figure 5A). In Condition 2, a small area in the left vSMG was activated (Figure 5B). In Condition 3, the bilateral vSMG, ventral part of the AG (vAG), and right pMTG were activated (Figure 6A). In Condition 4, the bilateral vSMG, bilateral pMTG, and right AG were activated (Figure 6B).

**Table 1:** Significantly activated cortical regions in the four conditions in group analyses using NIRS-SPM.

Condition	Hemisphere	MNI coordinates			*T*-value	BA	Cortical regions	
Direction of Rotation Visual stimulation		*X*	*Y*	*Z*			Anatomical	Functional
Condition 1 Rotation: R Visual stimulation: C	L	−65	−16	39	1.9523	3, 1, 2	PoG	S1
	R	68	−19	38	2.2803	40	vSMG	TPJ
		69	−15	31	1.6133	3, 1, 2	PoG	S1
Condition 2 Rotation: L Visual stimulation: C	L	−68	−27	33	1.8313	40	vSMG	TPJ
Condition 3 Rotation: R Visual stimulation: I	L	−68	−27	33	2.7574	40	vSMG	TPJ
	R	62	−63	21	2.0978	39	vAG	TPJ
		70	−35	29	1.6982	40	vSMG	TPJ
		49	−79	14	1.7484	19	pMTG	hMT+
Condition 4 Rotation: L Visual stimulation: I	L	−69	−33	26	2.6512	40	vSMG	TPJ
		−59	−70	9	1.9816	19	pMTG	hMT+
	R	70	−26	33	2.7709	40	vSMG	TPJ
		58	−62	36	1.862	39	AG	TPJ
		54	−75	22	2.0687	19	pMTG	hMT+

*R, right; L, left; C, congruent; I, incongruent; BA, Brodmann area; PoG, postcentral gyrus; vSMG, ventral part of the supramarginal gyrus; pMTG, posterior part of the medial temporal gyrus; vAG, ventral part of the angular gyrus; S1, primary somatosensory area; TPJ, temporoparietal junction; hMT+, human MT+ complex*.

### Correlation Between Hemodynamic Activity and Subjective Vertiginous Sensation

**Figure 7:**
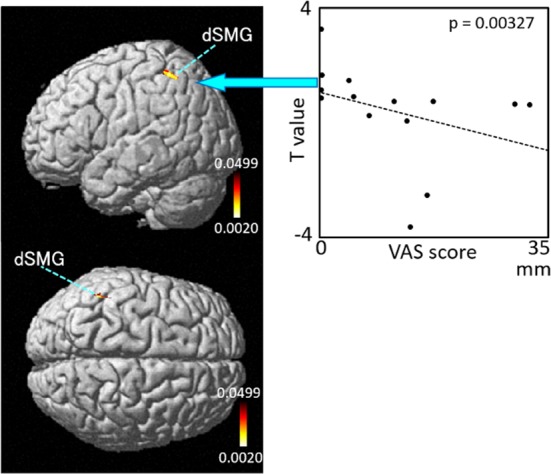
Relationships between cortical hemodynamic activity (*T*-values) and subjective vertiginous sensation (VAS scores) in Condition 1. Left panel indicates statistical maps of the brain regions with significant correlation between cortical hemodynamic activity (*T*-value) and subjective vertiginous sensation (VAS score). A negative correlation is found in the left dorsal part of the SMG (dSMG) in Condition 1. Right panel indicates an example of the scatter plots (Spearman’s rank coefficient test, *p* = 0.00327).

**Figure 8:**
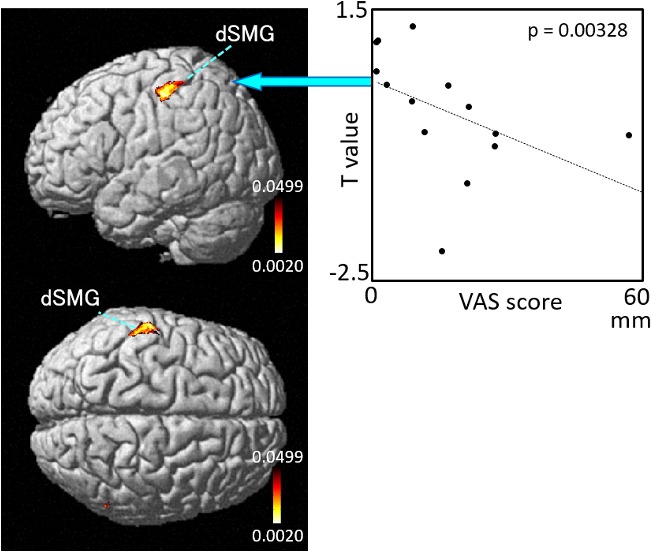
Relationships between cortical hemodynamic activity (*T*-values) and subjective vertiginous sensation (VAS scores) in Condition 3. The left panel indicates statistical maps of the brain regions with a significant correlation between cortical hemodynamic activity (*T*-value) and subjective vertiginous sensation (VAS score). A negative correlation is found in the left dorsal part of the SMG (dSMG) as indicated on the scatter plot for Condition 3. The right panel indicates an example of the scatter plots (Spearman’s rank coefficient test, *p* = 0.00328).

**Figure 9:**
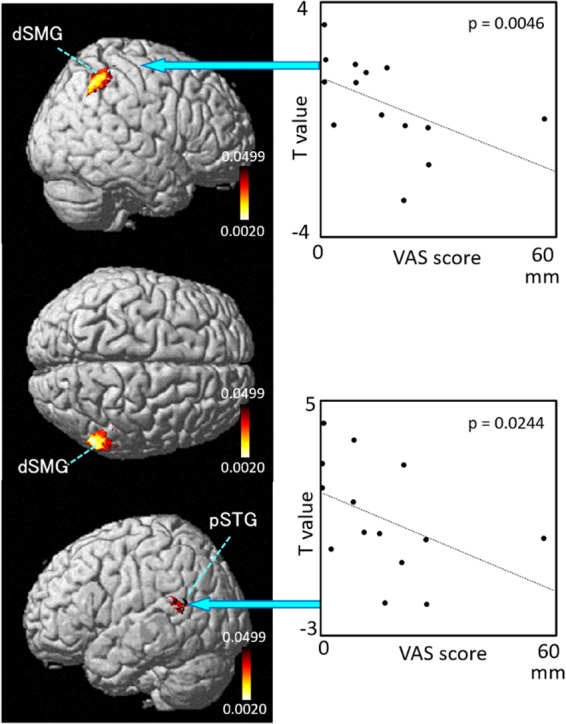
Relationships between cortical hemodynamic activity (*T*-values) and subjective vertiginous sensation (VAS scores) in Condition 4. The left panel indicates statistical maps of the brain regions with a significant correlation between cortical hemodynamic activity (*T*-value) and subjective vertiginous sensation (VAS score). A negative correlation is found in the right dorsal part of the SMG (dSMG) and left posterior part of the superior temporal gyrus (pSTG) in Condition 4. The right panel indicates examples of the scatter plots in the dSMG (Spearman’s rank coefficient test, *p* = 0.0046) and pSTG (Spearman’s rank coefficient test, *p* = 0.0244).

The results of the correlation analyses based on Spearman’s rank coefficient test of the 14 subjects’ data are shown in Figures 7–9 (side and top views). The pixels with significant correlation (i.e., *p* < 0.05) are colored on the standard brain. The statistical results are also listed in Table 2. A negative correlation between *T*-values and subjective vertiginous sensation was found in the dorsal part of the left dSMG in Condition 1 (*p* = 0.00327; Figure 7) and Condition 3 (*p* = 0.00328; Figure 8). In Condition 4, a negative correlation was found in the right dSMG (*p* = 0.0049) and posterior part of the left STG (Figure 9). No significant correlation was found in Condition 2.

**Table 2:** Summary of the correlation analyses between cortical hemodynamic responses (*T*-value) and subjective vertiginous sensation (VAS score).

Hemisphere	Cortical regions	BA	Condition 1 Rotation: R Visual: C	Condition 2 Rotation: L Visual: C	Condition 3 Rotation: R Visual: I	Condition 4 Rotation: L Visual: I
Right	dSMG	40				Negative
	pSTG	22				
Left	dSMG	40	Negative		Negative
	pSTG	22				Negative

*Negative, negative correlation; R, right; L, left; C, congruent; I, incongruent; BA, Brodmann area; dSMG, dorsal part of the supramarginal gyrus; pSTG, posterior part of the superior temporal gyrus*.

## Discussion

The present study indicated that sensory conflict strongly increased hemodynamic activity in a wide area including the bilateral vSMG and pSTG, which is called the TPJ, and pMTG. However, small activations in the bilateral primary somatosensory areas and vSMG were found under the congruent visual stimulation. Thus, the results indicated that sensory conflict between the visual and horizontal rotatory vestibular stimulations activated the bilateral TPJ and pMTG. In contrast, hemodynamic activity in the bilateral dSMG in and around the IPS was negatively correlated with subjective vertiginous sensation. These cortical regions are key structures of the cortical network for self-motion perception and visual–vestibular integration (Billington and Smith, 2015; Smith et al., 2017; Cheng and Gu, 2018).

### Activation of the pMTG

In the present study, hemodynamic activity increased in the pMTG under incongruent visual stimulation (Conditions 3 and 4). The results suggest that these cortical regions are involved in information processing of sensory conflict between visual and rotatory vestibular stimulation. These cortical regions observed in our study may be homologous to the human MT+ complex, which are motion-sensitive visual areas and typically found on the bank of a limb of the inferior temporal sulcus (Huk et al., 2002). The human MT+ complex has been hypothesized to be homologous to those of monkeys and has two subdivisions, the MT and the MST (Huk et al., 2002). In monkeys, dorsal MST neurons responded to both optic flow and translational movement (Duffy, 1998; Angelaki et al., 2011), suggesting MST involvement in the integration of visual and vestibular information in self-motion perception (Angelaki et al., 2011). Furthermore, monkey dorsal MST neurons preferentially responded to rotation with incongruent visual and vestibular inputs (Takahashi et al., 2007). In a human fMRI study, vestibular stimulation by galvanic vestibular stimulation activated the MST in the visual cortical areas in darkness, suggesting that visual and vestibular convergence might occur in the human MST during self-motion (Smith et al., 2012). Recent connectivity analyses using fMRI showed that the human MST may act as the relevant mediating network hub for the processing of conflicting visual–vestibular motion information (Rühl et al., 2018). These results suggest that the bilateral pMTG activated in the present study might correspond to the human MT+ complex, especially the human MST, and might be involved in detection of sensory conflict between visual and vestibular stimulations.

### Activation of the TPJ

In the present study, bilateral activation of the vSMG, vAG, and pSTG around the limb of the Sylvian fissure was found in Conditions 3 and 4 with incongruent visual and vestibular stimulations. These areas correspond to the TPJ that surrounds the human homolog of the monkey PIVC (the PIVC in humans) in the mid-posterior Sylvian fissure (Lopez and Blanke, 2011; Frank and Greenlee, 2018). The TPJ is a large region including the pSTG, AG, SMG, and the parietal operculum (Pfeiffer et al., 2014) and receives outputs from the PIVC involved in visual–vestibular processing (see below).

The PIVC in humans is strongly interconnected with other vestibular cortical areas and is hypothesized to be a core region for vestibular information processing (Brandt and Dieterich, 1999; Eickhoff et al., 2006). Extensive functional imaging studies on vestibular processing suggest that the PIVC in humans spanning to the TPJ is a multisensory region that receives not only vestibular but also visual or somatosensory inputs (Lobel et al., 1998; Bense et al., 2001; Bottini et al., 2001; Deutschländer et al., 2002; Fasold et al., 2002; Dieterich et al., 2003; Eickhoff et al., 2006; Dieterich and Brandt, 2008; zu Eulenburg et al., 2012; Bzdok et al., 2013).

A recent review article suggests that the PIVC reported in previous human imaging studies on vestibular processing contains two separate areas: the PIVC located in the parietal operculum and the posterior insular cortex (PIC) located in the retroinsular cortex (Frank and Greenlee, 2018). The authors named these two regions “PIVC+” as they are similar in some regard (both respond to vestibular stimuli) but dissimilar in others (PIVC is suppressed during visual processing, whereas the PIC is strongly activated; Frank et al., 2014, 2016; Frank and Greenlee, 2018). Recent fMRI studies using simultaneous visual (horizontal optokinetic stimulation) and vestibular (caloric irrigation or voluntary head rotation) stimulations reported activation in the PIC in incongruent visual–vestibular stimulation, suggesting that the PIC is involved in integration and disambiguation of visual–vestibular cues (Roberts et al., 2017; Schindler and Bartels, 2018). The PIVC may encode head and full-body movements and is involved in the estimation of heading direction through such movements, whereas the PIC may be involved in the estimation of heading direction by combining visual and vestibular cues and distinction between visual self-motion and visual object motion, which may be supported by neurons with incongruent visual–vestibular tuning (see a review by Frank and Greenlee, 2018).

The outputs of visual–vestibular processing from the PIVC+ are sent to the TPJ (Frank and Greenlee, 2018). fNIRS studies during postural balancing using the CDP also reported that the TPJ was activated in sensory conflict among vestibular, visual, and somatosensory inputs, consistent with the present results (Karim et al., 2013; Takakura et al., 2015; Lin et al., 2017).

It has been proposed that the vestibular system, especially the TPJ, is essential for representation of spatial aspects of bodily self-consciousness (Pfeiffer et al., 2014). Furthermore, a previous fMRI study using virtual reality reported that activity of the TPJ was associated with the sense of changes in self-location induced by incongruent visual–tactile stimulation (Ionta et al., 2011). These findings suggest that bilateral TPJ activation in the present study might reflect altered perception of head position and movements and neural process for an egocentric representation of the self in space in incongruent visual–vestibular conditions.

### Correlation Between Hemodynamic Activity in the dSMG and Subjective Vertiginous Sensation

In the present study, hemodynamic activity in the bilateral dSMG in and around the IPS was negatively correlated with subjective vertiginous sensation. The IPS is implicated in sensorimotor integration. In monkeys, the VIP, located in the fundus of the IPS, receives multimodal information: (1) vestibular information from the PIVC (Guldin et al., 1992; Lewis and Van Essen, 2000); (2) vestibular and somatosensory information from the vestibular neck subregions in areas 3a and 2 (Guldin et al., 1992; Lewis and Van Essen, 2000); (3) visual information from the medial temporal (MT) and MST complex (Lewis and Van Essen, 2000); and (4) somatosensory information from the S1 area (Lewis and Van Essen, 2000). In humans, the IPS is involved in the representation of coherent body images during sensory stimulation of multimodal stimuli in incongruent (Hagura et al., 2007; Bufalari et al., 2014) and congruent (Ehrsson et al., 2004; Petkova et al., 2011) conditions.

The negative correlation between the hemodynamic activities in the dSMG and subjective vertiginous sensation indicates that greater activity in the dSMG induces a weaker subjective vertiginous sensation during visual–vestibular sensory conflict. This suggests that the subjects are heterogeneous in sensory reweighting during sensory conflict. A neurophysiological study reported that monkey VIP neurons represented vestibular heading in an egocentric (body-centered) reference frame in a body-fixed gaze condition that corresponds to the present experimental situation (Chen et al., 2018). The subjects with higher dSMG activity and less vertiginous sensation might tend to represent the body in an egocentric reference frame. That is, under the incongruent condition, the sensory weight of visual information might be decreased in these subjects, which leads to higher weight of the vestibular system as the reliable source of information, which might result in flexible transformation of the spatial reference frame to an egocentric (body-centered) one. Consistent with this idea, a computational model suggests that different reference frames could be used based on the agent’s reliance in a specific reference frame and that the frame frequently switches between them (Oess et al., 2017). The subjects, who could not flexibly switch reference frames during sensory conflict, might feel a vertiginous sensation.

### Possible Clinical Application of fNIRS

fNIRS has several advantages compared with other neuroimaging modalities such as fMRI, MEG, and PET. In particular, fNIRS can make measurements without preventing bodily movements, and the present apparatus is highly portable, being suitable for all possible subject populations from newborns to the elderly and for various experimental settings, both inside and outside the laboratory (Pinti et al., 2020). We could measure the cortical hemodynamic responses to rotatory stimulation using two portable NIRS systems mounted on the rotatory chair. The paradigm used in the present study has been used for clinical examination of patients with vertigo in general. The present results suggest that fNIRS could be applied to clinical use for simultaneous recording of cerebral hemodynamic activity and peripheral vestibular functions.

Furthermore, the present results indicated that hemodynamic activity in the dSMG adjacent to the IPS was negatively correlated with subjective vertiginous sensation. Recent studies reported that various neurofeedback therapies using NIRS were effective in patients with stroke (Mihara and Miyai, 2016), social anxiety disorder (Kimmig et al., 2018), and attention-deficit/hyperactivity disorder (Blume et al., 2017). These findings suggest that real-time neurofeedback training using fNIRS to increase hemodynamic activity in the dSMG adjacent to the IPS could be effective to treat motion sickness, visual vertigo, or intractable chronic dizziness such as persistent postural perceptual dizziness.

### Limitations

There are several limitations in this study. First, the spatial resolution of NIRS is lower than that of fMRI, and it could not measure hemodynamics in the deeper brain regions including the insula, opercular regions, cerebellum, basal ganglia, or hippocampus. Furthermore, recorded cortical regions were limited in the present study: we recorded only the bilateral temporoparietal areas, whereas most parts of the frontal and parietal cortices were not measured. Further studies are required to investigate the roles of these brain regions in sensory conflict.

Second, the group statistical analyses of Deoxy-Hb signals did not indicate significant changes in the present study. As Oxy-Hb signals are more susceptible to systemic changes in blood circulation than are Deoxy-Hb, Oxy-Hb signals could yield false-positive data (Tachtsidis and Scholkmann, 2016). However, the signal-to-noise ratio of Deoxy-Hb signals is lower than that of Oxy-Hb signals (Sato et al., 2016), and Deoxy-Hb signals are sensitive not only to venous blood oxygenation but also to venous blood volume (Hoshi et al., 2001). Consequently, the direction of changes in Deoxy-Hb signals was variable across tasks and individuals, whereas the direction of changes in Oxy-Hb signals was consistent (Hoshi et al., 2001; Toichi et al., 2004; Sato et al., 2005). On the basis of these findings, we estimated Oxy-Hb data as cerebral hemodynamic activity in the present study. Future studies, which incorporate methods such as principal component spatial filtering to separate cerebral hemodynamic activity from the systemic component (Zhang et al., 2017), should be considered.

Third, we could not put foam rubber sheets between the rotatory chair and each subject’s hip or back to reduce somatosensory inputs because we had to hold the subject’s body tightly in the chair to prevent it from falling during rotation. Furthermore, we could not use ear plugs or noise-cancelling earphones to reduce auditory sounds (e.g., mechanical or wind noises) as we had to announce the timing of opening and closing of the subjects’ eyes by sound cues. Thus, the subjects could use these other inputs to recognize their orientation in space, and thus, we cannot completely deny the effect of sensory inputs other than visual and vestibular inputs (e.g., auditory and somatosensory inputs) during the rotatory task. However, previous studies reported that auditory information seems not to affect head postures (Thomas et al., 2018) and that trunk tactile cues did not affect the subjective sensation of rotation (Cheung and Hofer, 2007). Although the effects of these other sensory inputs seem to be low, further studies are required to assess the effects of the other sensory inputs on vertiginous sensation.

Fourth, the activated areas in the MST showed non-Gaussian distributions in Conditions 3 and 4. These regions were located in the posterior and inferior borders of the recording areas. Normalization of NIRS channel locations in individual subjects based on the MNI brain resulted in deviation of the NIRS channels from the MST in 3–4 subjects. The non-Gaussian distributions in and around the MST might be ascribed to a smaller number of NIRS channels over the MST due to the deviation of the channels. Further studies with a larger number of NIRS channels to record wider cortical regions are required to investigate the activity in the MST during sensory conflict.

Fifth, optokinetic visual stimuli usually elicit nystagmus. Therefore, nystagmus could affect cortical activation during the visual and rotatory tasks. In the present study, although the same optokinetic stimulations eliciting nystagmus were used in the congruent and incongruent visual conditions, the cortical areas activated were different between the two conditions. This suggests that differences in the areas activated might be attributed to factors other than nystagmus. Furthermore, a previous study reported that nystagmus itself activated mainly area V6A in the medial parieto-occipital sulcus (Konen et al., 2005), which is located outside the cortical areas recorded in the present study. These findings suggest that the effects of nystagmus itself on cortical activation might be small in the present study.

## Conclusion

The present study indicated that sensory conflict in the incongruent visual–vestibular condition significantly increased hemodynamic activity in the bilateral pMTG (corresponding to human MT/MST) and TPJ. Human MT/MST and TPJ have been reported as the motion-sensitive visual cortex and vestibular cortices, respectively (Huk et al., 2002; Lopez and Blanke, 2011), but also receive multimodal information (Smith et al., 2012; zu Eulenburg et al., 2012; Bzdok et al., 2013; Pfeiffer et al., 2014). These findings suggest that human MT/MST and the TPJ might be crucial for the detection of sensory conflicts between visual and rotatory vestibular stimulations.

Furthermore, the hemodynamic activity in the dSMG in and around the IPS, which is implicated in the egocentric reference frame (Chen et al., 2018), was negatively correlated with subjective vertiginous sensation during the visual and vestibular stimulations. Activation of the dSMG in the subjects with less vertiginous sensation suggests both that these subjects might switch reference frames to an egocentric reference frame and that flexible changes in reference frames might be crucial to the reduction of subjective vertiginous sensation during sensory conflicts. Deficits in these flexible changes might induce motion sickness, visual vertigo, or persistent postural perceptual dizziness. Further studies are required to elucidate the neural mechanisms responsible for the flexible shift of spatial reference frames and subjective vertiginous sensation during sensory conflict.

## Data Availability Statement

The datasets generated for this study are available on request to the corresponding author.

## Ethics Statement

The studies involving human participants were reviewed and approved by the ethical committee of the University of Toyama. The patients/participants provided their written informed consent to participate in this study.

## Author Contributions

NN, HT, and HS designed this research. NN, HT, NU, and SI performed the research. NN, HT, MF, and KA analyzed the data. NN, HT, HS, and HN wrote the manuscript.

## Conflict of Interest

The authors declare that the research was conducted in the absence of any commercial or financial relationships that could be construed as a potential conflict of interest.

## Footnotes

^1^https://bispl.weebly.com/nirs-spm.html
